# Peripheral sensory neuron injury contributes to neuropathic pain in experimental autoimmune encephalomyelitis

**DOI:** 10.1038/srep42304

**Published:** 2017-02-09

**Authors:** I-Ching Wang, Chen-Yen Chung, Fang Liao, Chih-Cheng Chen, Cheng-Han Lee

**Affiliations:** 1 Institute of Biomedical Sciences, Academia Sinica, 128, Sec. 2, Academia Rd., Taiwan; 2 Department of Life Science, National Taiwan University, Taiwan

## Abstract

Multiple sclerosis (MS)-induced neuropathic pain deteriorates quality of life in patients but is often refractory to treatment. In experimental autoimmune encephalomyelitis (EAE), a rodent model of MS, animals develop neuropathy and inflammation-induced tissue acidosis, which suggests the involvement of acid-sensing ion channels (ASICs). Also, peripheral neuropathy is reported in MS patients. However, the involvement of the peripheral nervous system (PNS) in MS neuropathic pain remains elusive. This study investigated the contribution of ASICs and peripheral neuropathy in MS-induced neuropathic pain. Elicited pain levels were as high in *Asic1a*^−/−^, *Asic2*^−/−^ and *Asic3*^−/−^ mice as wild-type mice even though only *Asic1a*^−/−^ mice showed reduced EAE disease severity, which indicates that pain in EAE was independent of disease severity. We thus adopted an EAE model without pertussis toxin (EAEnp) to restrain activated immunity in the periphery and evaluate the PNS contribution to pain. Both EAE and EAEnp mice showed similar pain behaviors and peripheral neuropathy in nerve fibers and DRG neurons. Moreover, pregabalin significantly reduced neuropathic pain in both EAE and EAEnp mice. Our findings highlight the essential role of the PNS in neuropathic pain in EAE and pave the way for future development of analgesics without side effects in the CNS.

Multiple sclerosis (MS) is a demyelinating autoimmune disease causing motor deficit due to neuronal damage-induced inflammation and demyelination in the central nervous system (CNS)^1^,^2^. It is the most frequent debilitating disease in young adults. Among MS-associated symptoms, chronic neuropathic pain severely reduces quality of life. Pain is reported by approximately 50% of MS patients and can be reported by 90%^3^,^4^,^5^. Furthermore, MS-induced chronic pain is significantly associated with MS-related disability and depression^4^. Therefore, mitigating chronic neuropathic pain in MS patients can improve their quality of life. However, the underling mechanisms of MS-induced chronic pain remain unclear.

Acid-sensing ion channels (ASICs), which are activated in tissue acidosis conditions, participate in pain sensation^6^. Inflammation in MS is often accompanied by acidosis in the spinal cord, which may lead to activation of ASICs. Lars Fugger and colleagues found ASIC1a involved in disease development in experimental autoimmune encephalomyelitis (EAE)^7^, a well-established animal model of MS. However, the role of ASICs in MS-induced chronic pain is still unknown.

Pain-like behaviors have been explored in EAE, and they occur before the onset of motor deficit and autoimmune infiltration in the CNS^8^,^9^. In addition, these pain-like behaviors in EAE mice, similar to clinical administration, can be ameliorated by anticonvulsant and antidepressant drugs^8^,^9^,^10^,^11^,^12^,^13^. Hence, the EAE model is suitable for exploring the underlying molecular and pathophysiological mechanisms as well as developing more effective analgesic candidates in MS-associated chronic neuropathic pain.

Comprehensive investigation of the cellular and pathological mechanism of chronic pain development has elucidated the importance of peripheral sensory-neuron activity. For instance, studies of fibromyalgia-associated pain in humans and animal models indicate that peripheral administration of lidocaine, blocking local neuronal activity, can relieve mechanical hyperalgesia and allodynia^14^,^15^,^16^,^17^,^18^,^19^,^20^. Also, damaged peripheral sensory neurons are involved in neuropathic pain associated with diabetes, chemotherapy, post-herpetic neuroglia, radiculopathy, etc.^21^,^22^. However, whether peripheral sensory neurons contribute to MS-induced neuropathic pain is largely unknown.

Accumulating evidence has shown that MS-induced pathological and functional damage in the peripheral nervous system (PNS) may be involved in the development of neuropathic pain. First, biopsy specimens of sural nerves from MS patients showed higher frequency of internodes with at least 50% reduced myelin thickness^23^. Second, in humans and rodents, many myelin proteins, including myelin basic protein (MBP), myelin proteolipid protein, myelin oligodendrocyte glycoprotein (MOG) and galactocerebroside, thought to be exclusively expressed in the CNS, are expressed in the PNS, so autoimmunity may attack the PNS^24^,^25^,^26^,^27^,^28^. Third, a case report of an MS patient demonstrated PNS symptoms, including areflexia and specific nerve conductive features of inflammatory demyelinating polyneuropathy (CIDP), dominating the clinical features, without relapsing MS for at least 6 years^29^. This temporal and clinical association between MS and CIDP suggests that MS-induced neuronal damage in both the CNS and PNS might share the same insults from T-cell activation to target same myelin proteins. This evidence indicates that peripheral sensory-nerve injury may contribute to chronic pain development in MS.

Here we aimed to probe the neurobiological basis of EAE-induced chronic neuropathic pain. We first evaluated the role of ASICs in pain development and whether the PNS damage contributes to clinical and pathological deficits in EAE mice with or without pertussis toxin (PTX). Next, we examined the neuropathic injury of dorsal root ganglia (DRG) in EAE mice by staining cyclic AMP-dependent transcription factor-3 (ATF-3), a well-known neuropathic pain marker, then verified the identity of injured DRG neurons. Last, we aimed to determine whether the anticonvulsant drug pregabalin has an analgesic effect in EAE mice with or without PTX injection.

## Results

### All ASIC-subtype knockout mice elicit similar levels of EAE-induced pain to wild-type mice regardless of their different contributions to clinical severity

**Figure 1:**
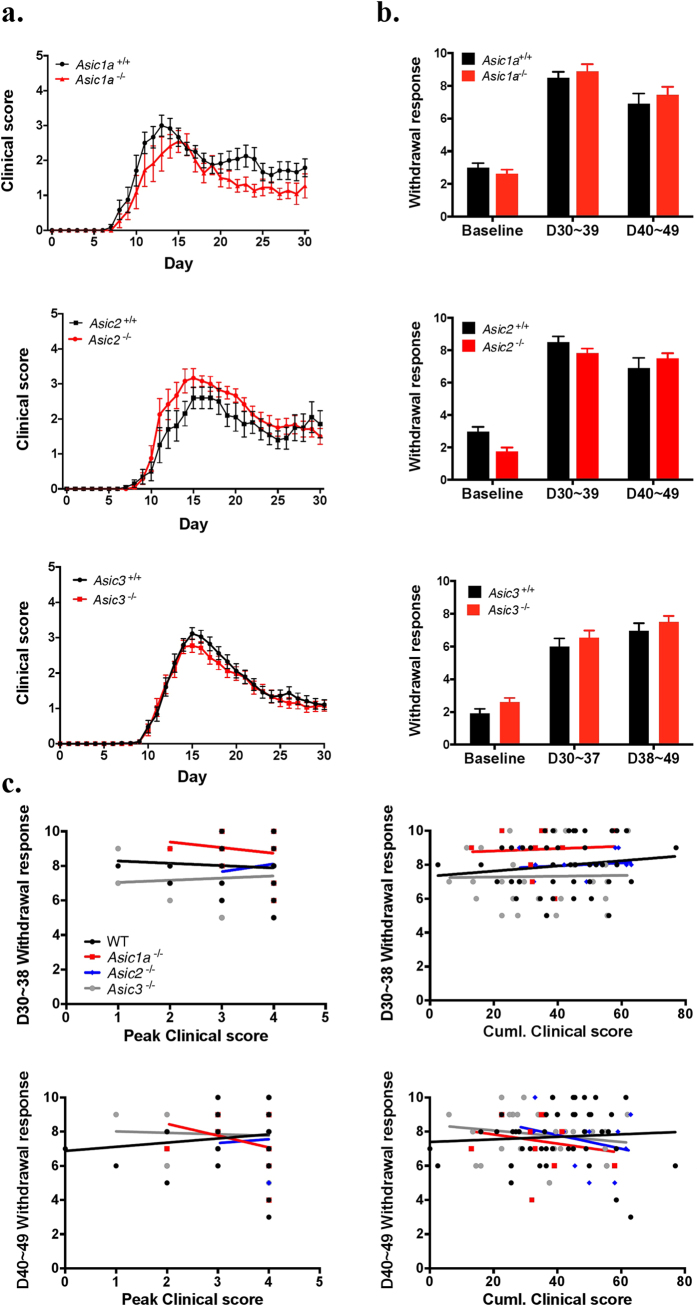
ASIC-knockout EAE mice showed pain comparable to the wild type despite different contribution to severity. (**a**) EAE was induced in *Asic1a*^−/−^ (N = 11), *Asic2*^−/−^ (N = 13), *Asic3*^−/−^ (N = 33) and littermate control *Asic1a*^+/+^ (N = 16), *Asic2*^+/+^ (N = 10), *Asic3*^+/+^ (N = 34) mice. Disease severity was monitored by daily clinical scoring for 30 days. (**b**) Mechanical sensitivity was assessed by von Frey test before EAE induction as baseline, 30 to 39 days post-induction and 40 to 49 days post-induction. (**c**) Regression line of peak score and cumulative score verses pain levels at 30 to 39 days and 40 to 49 days post-induction of EAE in wild-type, *Asic1a*^−/−^, *Asic2*^−/−^ and *Asic3*^−/−^ mice. Statistics were analyzed by two-way ANOVA. Genotype effect: *Asic1a*^−/−^: F_(1,21)_ = 5.421, P = 0.03; *Asic2*^−/−^: F_(1,20)_ = 1.778, P = 0.1974; *Asic3*^−/−^: F_(1,65)_ = 0.4818, P = 0.4901; Time effect: *Asic1a*^−/−^: F_(30,630)_ = 30.54, P < 0.001; *Asic2*^−/−^: F_(30,600)_ = 42.30, P < 0.001; *Asic3*^−/−^: F_(30,1905)_ = 120, P < 0.001. Interaction: no significance.

A previous study showed that *Asic1a* knockout ameliorated EAE-induced clinical deficits in mice, but its effect on pain was not explored^7^. Since EAE induced acidosis (pH 6.5~6.6) in the spinal cord, we tested whether specific ASIC subtype would affect the EAE-induced neuropathic pain. Here we conducted a comprehensive exam of EAE-induced neuronal disorders and pain in mice lacking *Asic1a, Asic2*, and *Asic3*. On analysis of clinical severity in EAE mice, *Asic1a*^−/−^ mice showed significantly milder disease progression than *Asic1a*^+/+^ mice (genotype: F_(1,21)_ = 5.4, P = 0.03). *Asic2*^−/−^ and *Asic3*^−/−^ mice showed no difference in disease progression as compared with wild-type littermates, although *Asic2*^−/−^ mice showed a trend to increased disease severity (F_(1,20)_ = 1.778, P = 0.19) (Fig. 1a). Previous studies demonstrated that ASIC2 expression could modulate the expression of ASIC1a^30^,^31^, so we performed western blot analysis to investigate the membrane proteins of DRG and found that loss of ASIC2 did not alter the expression of ASIC1a in DRG (See Supplementary Fig. S1 and Methods).

We next probed the effect of Asic knockout on EAE-evoked neuropathic pain. Since it has been reported that mechanical and thermal hyperalgesia is present in pre-onset of EAE mice^9^,^11^, we examined the mechanical and thermal hyperalgesia in different stages after EAE induction, including pre-onset (days 6 to 7), early onset (days 11 to 12), and recovery (days 30–37, 38–49). In both pre-onset and early-onset phases, mechanical and thermal sensitivity did not differ among Asic knockout groups as compared with the wild type (Supplementary Fig. S2). We found a significant interaction effect in mechanical hyperalgesia, and post-hoc analysis showed only *Asic1a*^−/−^ mice with lower mechanical responses to von Frey stimuli on days 6–7 than *Asic3*^−/−^ mice. On days 6–7, only *Asic3*^−/−^ mice showed significant mechanical hyperalgesia as compared with their pre-immune phase; the other groups of mice showed significant mechanical hyperalgesia on days 11–12. The onset of EAE-induced neuropathic pain differed greatly among genotypes and may be complicated by differential disease onset among mice. In the recovery phases, EAE induced mechanical hyperalgesia but not thermal hyperalgesia (Supplementary Fig. S3). All *Asic1a*^−/−^, *Asic2*^−/−^, and *Asic3*^−/−^ mice showed similar mechanical sensitivity as wild-type mice after EAE (Fig. 1b). The clinical deficits were not associated with levels of mechanical hyperalgesia in the recovery phase in EAE mice. Therefore, EAE disease severity may not be associated with pain.

To further demonstrate whether these two aspects are correlated with each other in EAE, we further analyzed the correlation between EAE-induced mechanical hyperalgesia and two parameters of clinical deficit: peak score and cumulative score (see Methods) and found no correlation with each other (Fig. 1c, Table 1). Therefore, EAE disease severity is independent of pain levels, which suggests EAE-induced motor deficits (clinical scores) and neuropathy pain develop by 2 independent mechanisms.

### EAEnp mice show increased mechanical hypersensitivity with almost normal mobility

Although most of the MS studies of EAE models focused on the CNS, whether the CNS or PNS contributes to EAE-induced neuropathic pain remains unclear. To investigate the mechanisms underlying EAE-induced neuropathic pain, we used an approach to separate the CNS and PNS effect. We used a modified EAE immunization model without PTX (named EAEnp), because PTX in EAE facilitates activated leukocyte infiltration into the CNS by increasing the permeability of the blood–brain barrier. In the EAEnp model, MOG-activated T cells are restrained in the periphery, so we could specifically explore the PNS effect.

**Figure 2:**
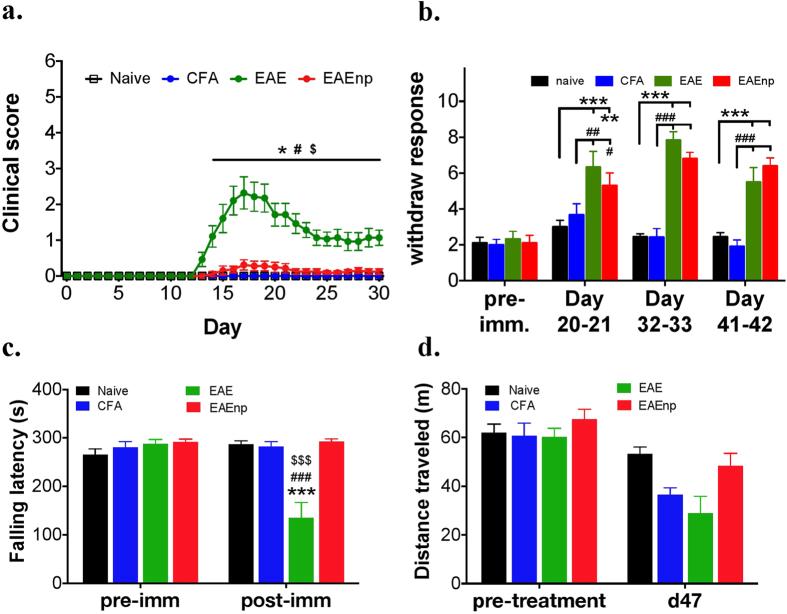
EAEnp mice developed pain without clinical deficits. (**a**) Clinical scores of mice in naive (N = 7), CFA (N = 7), EAE (N = 14), EAEnp (N = 16) groups at different days after immunization. Statistics were analyzed by two-way ANOVA (Interaction: F_(90,1200)_ = 10.08; Time: F_(30,1200)_ = 10.64; Treatment: F_(3,40)_ = 20.44). Post-Bonferroni analysis presented in the figure, *p < 0.05 vs. naive group; ^#^p < 0.05 vs. CFA group; ^$^p < 0.01 vs. EAEnp group. (**b**) von Frey test conducted at selected time points showing hyperalgesia in both EAE and EAEnp mice. Naive: N = 9, CFA: N = 12, EAE: N = 6, EAEnp: N = 10. Statistics were analyzed by two-way ANOVA (Interaction: F_(9,99)_ = 7.769, p < 0.001; Treatment: F_(3,33)_ = 30.03, p < 0.001; Time: F_(3,99)_ = 29.18, p < 0.001). Post-Tukey analysis presented in the figure, **p < 0.01, ***p < 0.001 vs. naive group; ^##^p < 0.01, ^###^p < 0.001 vs. CFA group. (**c**) Rota-rod test shows significantly shorter falling latency in EAE mice than other groups at 47 days post-immunization. Naive: N = 12, CFA: N = 11, EAE: N = 9, EAEnp: N = 11. Statistics were analyzed by two-way ANOVA (Interaction: F_(3,39)_ = 26.3; Time: F_(1,39)_ = 18.1; Treatment: F_(3,39)_ = 11.5). Post-Bonferroni analysis presented in the figure, ***p < 0.001 vs. naive group; ^###^p < 0.001 vs. CFA group; ^$$$^p < 0.001 vs. EAEnp group. (**d**) Open field test shows no difference in traveling distance between treatment groups.

We assessed the disease course in 4 experimental groups: naïve, CFA, EAE and EAEnp mice (Fig. 2a). EAE mice showed clinical deficits as previously reported, whereas EAEnp mice showed very mild clinical deficits. Mice injected with CFA showed no disease onset like naïve mice. During chronic pain assessment, EAE mice elicited hyperalgesia as was reported by previous studies, whereas EAEnp mice exhibited similar mechanical hypersensitivity as EAE mice at 20, 32–33 and 41–42 days after induction (Fig. 2b). Furthermore, EAE and EAEnp mice elicited similar mechanical hypersensitivity in the early-onset phase, whereas only EAE mice (but not EAEnp mice) showed decreased thermal response in the early-onset phase (Supplementary Fig. S4). In open-field and rota-rod tests, only EAE mice showed decreased locomotor activity in the rota-rod test, whereas mice in both CFA and EAEnp groups showed locomotor activity comparable to naïve mice (Fig. 2c,d). Thus, EAEnp mice showed elevated mechanical sensitivity without motor deficit, which suggests that the CNS damage may not be the direct cause of the neuropathic pain in EAE models.

### L4 spinal cord of EAEnp mice at disease peak shows no immune cells infiltrating and demyelination with very mild microglia activated in gray matter

**Figure 3:**
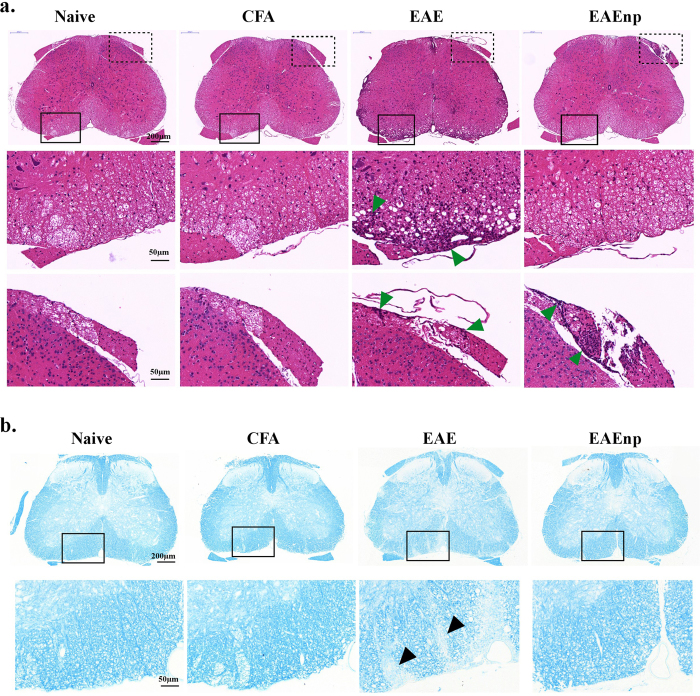
Immune-cell infiltration and demyelination occurred in EAE but not EAEnp mice. Lumbar spinal cord (L4) of each treatment was collected at the peak of disease (d15) and underwent staining. (**a**) H&E staining of L4 spinal cord. Magnified images of square areas in solid, the ventral horn, and dashed line, the dorsal root, are shown in the second and third rows, respectively. 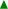, sites of immune-cell infiltration. (**b**) Luxol fast blue staining of L4 spinal cord. Magnified images of dashed line square are shown in the second row. ▴, sites of demyelination. Full-sized images and magnified images are in the same scale, respectively.

We next examined the pathological aspect of the CNS in the 4 groups of mice, focusing on infiltration of immune cells in the L4 spinal cord. On H&E staining, the L4 spinal cord of naïve and CFA mice showed little or no immune cell infiltration, whereas the EAE mouse spinal cord showed massive immune cell infiltration especially in the ventral horn (Fig. 3a). The spinal cords of EAEnp mice showed little immune cell infiltration similar to naïve or CFA mice. EAEnp mice showed accumulation of immune cells in the dorsal root, the sensory input from PNS, as did EAE mice (Fig. 3a).

Demyelination is another feature characteristic of EAE model. Here, Luxol fast blue staining in spinal cords of naïve and CFA mice demonstrated negligible demyelination, with EAE mice showing obvious demyelination (Fig. 3b). Notably, EAEnp mice showed no signs of demyelination.

**Figure 4:**
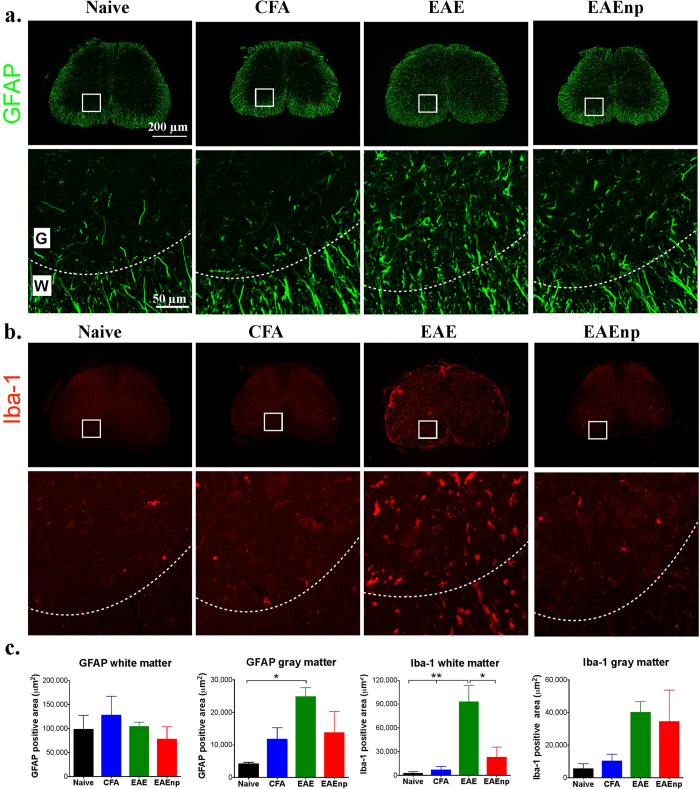
Astrocytes and microglia activation in the spinal cord observed in EAE mice. Lumbar spinal cord (L4) for each treatment was collected at the peak of disease (d15) and underwent immunofluorescence staining. Magnified images of squared area are shown in the second row of each staining. Dashed curves indicate the border between white matter and gray matter. (**a**) GFAP staining for astrocytes in each treatment. (**b**) Iba-1 staining of microglia in each treatment. (**c**) Quantification of GFAP and Iba-1 staining in white matter and gray matter. Statistics were analyzed by one-way ANOVA (GFAP white matter: F_(3,10)_ = 0.6455, p = 0.6033; GFAP gray matter: F_(3,10)_ = 3.998, p = 0.0414, Naive vs. EAE: p = 0.0427; Iba-1 white matter: F_(3,10)_ = 9.327, p = 0.0030. Naive vs. EAE: p = 0.0055, CFA vs. EAE: p = 0.0075, EAE vs. EAEnp: p = 0.0167; Iba-1 gray matter: F_(3,10)_ = 2.023, p = 0.1747). *p < 0.05, **p < 0.01. Full-sized images and magnified images are in the same scale, respectively. W: white matter; G: gray matter.

We further characterized the infiltrated cells by immunohistochemistry. Staining of the astrocyte marker GFAP in the L4 spinal cord revealed increased astrocyte number in EAE mice in gray matter as compared with other groups (Fig. 4a). Although astrocyte number was slightly increased in EAEnp gray matter, the result was not significantly different from other groups. Microglia was monitored by Iba-1 staining, and elevated microglia number was apparent in the L4 spinal cord in EAE mice (Fig. 4b). Quantitative analysis indicated significantly greater GFAP expression in L4 spinal-cord gray matter in EAE than naïve mice (Fig. 4c). Iba-1 expression was significantly elevated in EAE mouse L4 spinal-cord white matter as compared with other groups. Therefore, the spinal cord, representing the CNS, remained mostly undisturbed in EAEnp mice.

### Peripheral neuropathy after EAE or EAEnp induction

**Figure 5:**
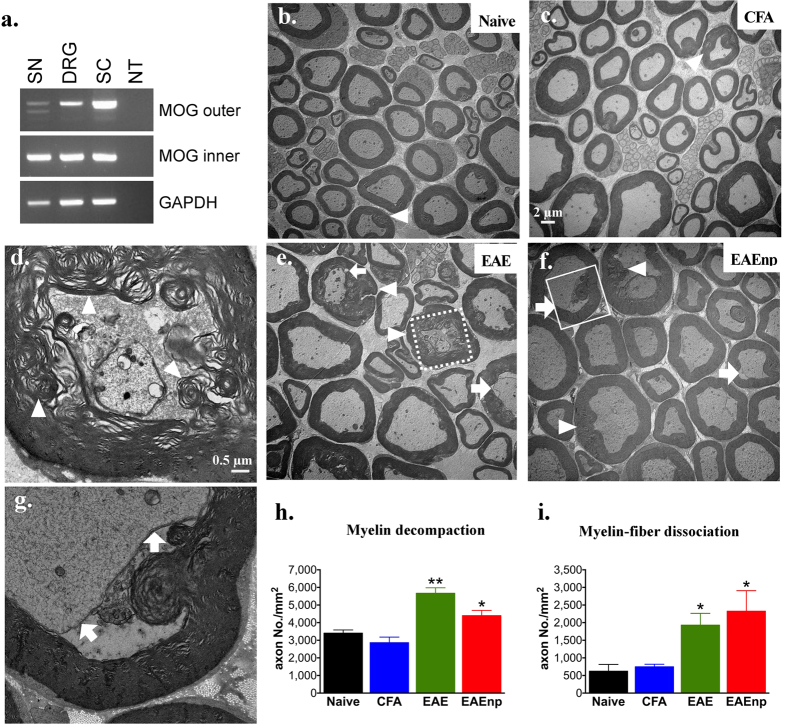
Myelin disruption shown in peripheral nerves in EAE and EAEnp mice. (**a**) Myelin oligodendrocyte glycoprotein (MOG) transcripts were detected by nested RT-PCR in sciatic nerve (SN), dorsal root ganglion (DRG), and spinal cord (SC) of naive wild-type mice. NT: negative control. (**b**,**c**,**e**,**f**) Sciatic nerves of each treatment were dissected at peak of disease (d15) for electron-microscopy image. (**d**) A representative image of an axon showing decompacted myelin in EAE, square area of dashed line in (**e**). (**g**) A representative image of an axon showing myelin-fiber dissociation in EAEnp, square area of solid line in (**f**). (**h**) Quantification of decompacted myelin. Statistics were analyzed by unpaired *t* test (EAE: p = 0.0022, EAEnp: p = 0.0437). (**i**) Quantification of myelin–fiber dissociation. Statistics were analyzed by unpaired *t* test (EAE: p = 0.0272, EAEnp: p = 0.0497). ⇧, axons with myelin–fiber dissociation. Δ, axons with decompacted myelin. *p < 0.05 vs. naive group. Naive N = 3, CFA N = 3, EAE N = 4, EAEnp N = 3. Scale in (**c**,**e**,**f**) is the same as (**b**). (**d**,**g**) are in the same magnification.

We next examined the possible contribution of the PNS to the EAE-induced neuropathic pain. MOG_35-55_ peptide, belonging to the CNS, has been widely used for EAE induction in mice; however, its expression in the PNS has not been confirmed. Here we showed MOG transcripts (493–667 bp) in mouse DRG and sciatic nerve (a representative peripheral nerve) by nested RT-PCR (Fig. 5a). To determine whether the expression of MOG in DRG could cause demyelination of peripheral nerves during EAE, we used electron microscopy to analyze the sciatic nerves from naïve, CFA, EAE and EAEnp mice. The myelin sheath of naïve and CFA mice was characterized by a regular and well-organized myelin sheath (Fig. 5b,c). However, the sciatic nerves of EAE and EAEnp mice showed a relatively decompacted myelin sheath, characterized by a dismantled myelin sheath (Fig. 5d,e,f). Also, many axons of EAE and EAEnp mice showed myelin-fiber dissociation, with the membrane of neurons dissociated from the myelin sheath (Fig. 5g). The frequency of decompacted myelin sheath or myelin-fiber dissociation was higher in axons of EAE and EAEnp than CFA and naïve mice (Fig. 5h,i). These results illustrate MOG expression in the PNS and myelin disruption in the PNS after EAE or EAEnp induction and suggest that the neuropathic pain induced in EAE results from peripheral neuropathy derived from demyelination.

### Injured DRG neurons are identified in EAE and EAEnp mice and partially characterized as myelinated or non-peptidergic neurons

**Figure 6:**
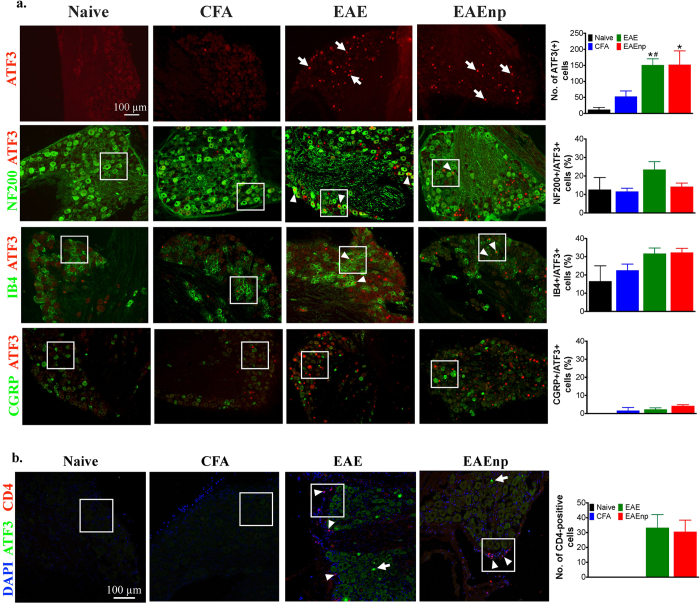
Characterization of ATF3-positive neurons and infiltrating T-helper cells in EAE and EAEnp mice. DRG at the 4^th^ lumbar segment was isolated from mice of each treatment at disease peak (d15). (**a**) DRG sections were stained with ATF3 to assess nerve injury. Characterization of injured neurons was assessed by co-staining ATF3 with different neuron markers. NF-200, neurofilament-200 (a marker of myelinated neurons); IB4, isolectin-B4 (a marker of non-peptidergic neurons); CGRP, calcitonin gene-related peptide (a marker of peptidergic neurons). ⇑, ATF3-positive neurons. Δ, co-localization of ATF3 with each neuron marker. Quantitative analysis of ATF-positive neurons and their subtypes in different treatment groups are shown in the right column. Statistics were analyzed by one-way ANOVA, *p < 0.05 vs. naïve group. ^#^p < 0.05 vs. CFA group. All images are in the same scale as indicated in ATF3 staining of Naïve group. b. DRG sections were stained with ATF3 and CD4 to assess the anatomical distribution of injured neurons and infiltrating T-helper cells. ⇑, ATF3-positive neurons. Δ, CD4-positive cells. Quantitative analysis of CD4-positive cells is indicated in the right column. Magnified images of square area are shown in Supplementary Fig. S6.

To better understand whether the PNS is involved in EAE-induced neuropathic pain, we examined the expression of activating transcription factor (ATF3), a commonly used neuron injury marker and related to neuropathic pain^20^,^32^, in the fourth lumbar DRG (L4 DRG) of naïve, CFA, EAE and EAEnp mice. ATF3-positive neurons were observed in L4 DRG neurons from both EAE and EAEnp mice and were absent in naïve and CFA mice (Fig. 6, Supplementary Fig. S5). Next, we further characterized the ATF3-positive DRG neurons (Fig. 6a; See Supplementary Fig. S6 for large magnification). DRG neurons can be approximately characterized as large-diameter, myelinated neurons that express NF-200 and small-diameter, non-myelinated neurons, which mainly encode nociceptive signals and can be divided into isolectin B_4_ (IB_4_)-positive non-peptidergic and IB_4_-negative peptidergic neurons^33^. The peptidergic population expresses neuropeptides such as substance P and calcitonin gene-related peptide (CGRP). About 20% of ATF3-positive DRG neurons were NF-200–positive and about 30% of ATF3-positive DRG neurons were IB_4_–positive in EAE mice. In EAEnp mice, NF-200 and IB_4_–positive cells accounted for 10% and 30% of ATF3-positive DRG neurons respectively. However, less than 5% of the ATF3-positive neurons were CGRP-positive in EAE and EAEnp mouse L4 DRG. CD4-positive T lymphocytes were present in L4 DRG of EAE and EAEnp mice but not DRG of naïve and CFA mice (Fig. 6b). The cell type of CD4-positive T lymphocytes was mainly Th1 cells, which express interferon γ but not interleukin 17 (Supplementary Fig S7 and Methods). Our results elucidate the EAE-induced neuronal injury, which might be associated with CD4-positive cell infiltration, in DRG neurons, containing myelinated neurons and non-peptidergic neurons.

### EAE- and EAEnp-induced mechanical hyperalgesia was ameliorated by pregabalin

**Figure 7:**
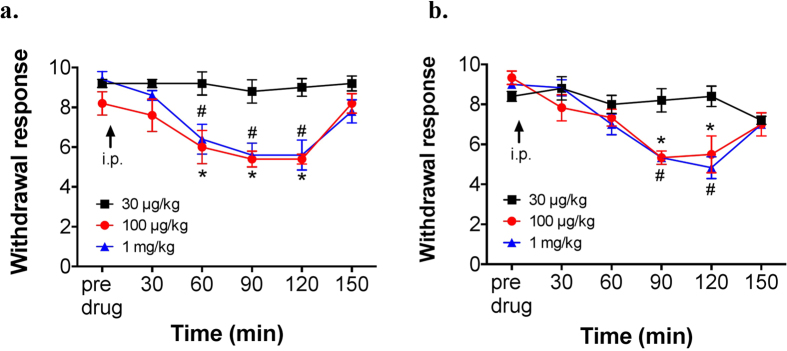
Analgesic effects of pregabalin in EAE and EAEnp mice. Mechanical sensitivity to a 0.02-g von Frey filament was evaluated in mice at 41 to 50 days post-immunization. (**a**) Dose-dependent effect of pregabalin in EAE mice (30 μg/kg N = 5, 100 μg/kg N = 5, 1 mg/kg N = 5, i.p.). Statistics were analyzed by two-way ANOVA (EAE: Interaction: F_(10,60)_ = 3.264; Time: F_(5,60)_ = 14.77; Drug dose: F_(2,12)_ = 12.35). (**b**) Dose-dependent effect of pregabalin in EAEnp mice (30 μg/kg N = 5, 100 μg/kg N = 6, 1 mg/kg N = 6, i.p.). Statistics were analyzed by two-way ANOVA (EAEnp:Interaction: F_(10,70)_ = 4.121; Time: F_(5,70)_ = 17.53; Drug dose: F_(2,14)_ = 5.176). Post-Bonferroni analysis presented in the figure, *P < 0.05, 30 μg/kg vs. 100 μg/kg; ^#^P < 0.05, 30 μg/kg vs. 1 mg/kg.

Gabapentinoids (e.g., gabapentin, pregabalin) have been recommended and found effective in ameliorating pain associated with MS in humans and rodents^34^,^35^. Hence, we tested whether pregabalin has an analgesic effect in EAE and EAEnp mice. Pregabalin showed similar dose-dependent analgesic effects in both EAE and EAEnp mice, with effective concentrations of 100 μg/kg and 1 mg/kg (i.p.) (Fig. 7). Hence, both EAE and EAEnp models may induce neuropathic pain via a shared pathway, the peripheral neuropathy.

## Discussion

In this study, we showed that ASIC1a is involved in EAE-induced motor deficits, but the channel was not associated with the development of neuropathic pain. Using a modified EAE model without PTX, EAEnp, we demonstrated that EAEnp mice elicit neuropathic pain without immune cells infiltrating in CNS, which suggests the contribution of the PNS in EAE-induced neuropathic pain. The neuropathic pain in EAE mice may be partly attributable to myelinated peripheral nerve injury as well as IB4-positive non-peptidergic nociceptors. Together, the current work highlights a specific type of peripheral neuropathy contributing to the development of MS-associated chronic pain and thus provides a new direction to treat the intractable pain.

ASIC1a is known to play an important role for sensing acidosis during ischemic stroke, seizure, fear, panic, and MS, as well as synaptic transmission^7^,^36^,^37^,^38^,^39^,^40^. A previous study also showed that blockade of ASIC1a activity would result in activation of the endogenous enkephalin pathway in the CNS and thus produce analgesia for neuropathic pain^41^. We were surprised to find normal development of EAE-induced chronic neuropathic pain in *Asic1a*^−/−^ mice, although they showed milder EAE clinical scoring than wild-type mice. This result excludes a role for ASIC1a in modulating EAE-induced pain and also raises a question of the CNS contribution to the pain development. Although the MS-associated pain is complex and we cannot totally exclude the contribution of the CNS, probing a possible role for the PNS in EAE-induced neuropathic pain is of interest.

Despite numerous studies of MS pain, evidence regarding the PNS contribution is scarce because of the limited diagnostic methods. Pollock and colleagues reported that many MS patients have abnormal sensory nerves, with internodes showing at least 50% reduction in myelin thickness^23^. In 2011, Gartzen *et al*. used electrophysiological diagnosis to investigate the PNS effect on MS pain and found that about 30% of MS patients showed lower conduction velocity in peripheral myelinated nerve fibers, despite no abnormalities in pain-related evoked potentials^42^. Ayromlou *et al*. further analyzed nerve electrophysiological properties of motor and sensory fibers in MS patients and found subtle sensory fiber abnormality^43^. Although diagnostic methods are insufficient to clearly and entirely investigate the peripheral sensory fiber function, these studies indicate that peripheral sensory neurons might be injured in MS patients and contribute to sensory illness.

However, previous studies using EAE models to investigate the neuropathic pain mechanism in MS revealed the involvement of both neurological and immunological factors in the PNS, including satellite glia-cell activation^44^ and elevated levels of tumor necrosis factor α^45^ and brain-derived neurotrophic factor^46^, in DRG of EAE animals. Moreover, our EAEnp mouse model is supported by evidence of mild symptoms accompanied by hyperalgesia in the rat EAE model, which also does not contain PTX^13^,^47^. To our knowledge, we are the first to use the EAEnp mouse model to investigate pain mechanisms in EAE and show that it is feasible in behavioral or pathological experiments.

We found immune cells infiltrating the dorsal roots of both EAE and EAEnp mice (Fig. 3a), which suggests that activated immune cells might infiltrate the spinal cord through dorsal roots. This observation might explain why approximately 25% of EAEnp mice showed mild disease signs such as tail weakness, although immune cell infiltration to the spinal cord was rarely found in EAEnp mice. Nevertheless, 75% of our EAEnp mice showed mechanical hypersensitivity without any clinical deficit and motor function interference. Hence, the EAEnp model is suitable and accurate for assessing neuropathic pain responses in EAE mice regardless of motor impairment during disease.

Spinal cord immune-cell infiltration and demyelination is characteristic of the EAE model but is absent in EAEnp mice. Thus, peripheral nerve insult by MOG-triggered autoimmunity may play an important role in EAE-induced neuropathic pain. Although MOG is predominantly expressed in the CNS, evidence of MOG mRNA expression in the PNS has been shown in primates and rats^48^ and in mice in our study. In the EAEnp mice, MOG-specific T cells might be trapped in the PNS and related to ATF3 activation in DRG neurons. A recent study also showed that PTX injection alone could induce ATF3 expression in DRG neurons in mice, but the ATF3 expression was much lower in PTX-only group than in EAE mice^49^. Comparing a previous study^49^ with our results of EAEnp mice, peripheral nerve injury in EAE might only partially result from PTX injection. Of note, previous studies used a rat EAE model, which did not include PTX injection, to study pain^13^,^47^. In addition to rats, human studies have also illustrated peripheral nerve injury in MS patients^23^. Together with data from the EAEnp model, we demonstrate that PNS is important for EAE neuropathic pain.

DRG is a highly heterogeneous tissue, composed of different types of sensory neuron populations. Each population may refer to a specific sensory pathway. Thus, characterization of the injured neurons in EAE may further reveal the possible factors involved in the neuropathic pain development. Here we identified that EAE-induced injured neurons were mainly NF-200–positive myelinated neurons (~20%) and IB4–positive non-peptidergic neurons (~30%), whereas CGRP-positive peptidergic neurons were almost never injured. NF-200–positive neurons are myelinated A fibers, including A-δ fibers that transmit acute pain and innervate in the lamina I, II outer and V parts of the spinal cord dorsal horn^33^,^50^. Previous studies showed that A-fiber demyelination would induce chronic neuropathic pain. Demyelination-induced loss of conduction in A fibers would result in plastic changes of C-polymodal nociceptors, which would generate spontaneous activity and hypersensitivity to innocuous stimuli^51^. IB4-positive neurons encode unmyelinated non-peptidergic C fibers that innervate into the lamina II inner layer and transmit neuropathic pain due to neuron injury^33^,^52^,^53^,^54^. In many neuropathic pain models, increased ATF3 expression is often coupled with upregulation of P2X3 in IB4-positive neurons^55^,^56^. However, CGRP-positive neurons are unmyelinated peptidgeric C fibers that innervate into the lamina I and II outer layers of the spinal cord dorsal horn and are involved in inflammatory pain^33^,^53^,^54^,^57^. Although NF-200−, IB4−, and CGRP-positive neurons represent almost >95% of DRG neurons^54^,^58^,^59^,^60^, more than 40% of ATF3-positive DRG neurons are not in these populations. Further studies should focus on the identity of these ATF-positive DRG neurons, to determine a full picture of EAE-induced peripheral neuropathy. Candidate markers for the non-NF-200, non-IB4, and non-CGRP DRG neuron populations may include galanin, neuropeptide Y, nitric oxide synthase, and lysophosphatidic acid receptor^33^,^61^,^62^.

Pharmacological studies of MS neuropathic pain treatment have revealed a limited effect of current analgesics such as morphine^63^. Anti-epileptic drugs such as gabapentin and pregabalin are some of the common drugs prescribed for MS pain in clinical practice^35^. Clinical research has indicated the effects of gabapentin and pregabalin in relieving pain in MS patients. Thus, we assessed the analgesic effect of pregabalin in EAE and EAEnp mice and found that it could alleviate pain in both models at a relatively high dose. Pregabalin is known to relieve neuropathic pain by reducing calcium influx and neurotransmitter release in the spinal cord by inhibiting the α2δ-1 subunit of T-type calcium channel trafficking to sensory nerve terminals^64^,^65^. Together with our results for EAE and EAEnp mice, pregabalin eliciting anti-nociception through the periphery can further support our hypothesis that PNS is involved in EAE neuropathic pain. Thus, we demonstrate the predicted validity of using the EAEnp model to explore the EAE-induced neuropathic pain and highlight the involvement of peripheral neuropathy in the development of MS-related pain.

## Methods

### Mice

*Asic1a* knockout (*Asic1a*^−/−^) mice were a kind gift from Dr. Chen-Chang Lien of National Yang-Ming University^66^. *Asic2* knockout (*Asic2*^−/−^) mice were purchased from the Jackson Lab. *Asic3* knockout (*Asic3*^−/−^) mice were generated as previously described^67^. All mutant mouse lines were congenic after backcrossing to C57BL6/J for at least 10 generations. Wild type and knockout mice were littermates from heterozygote breeding. All experiments involved 8- to 12-week-old female mice housed in a group (3–5) at a temperature-controlled environment with a 12-hr light/dark cycle from 8:00 to 20:00. All procedures followed the Guide for the Care and Use of Laboratory Animals published by the US National Institutes of Health (NIH publication No. 85-12, revised 1996) and approved by the Institutional Animal Care and Utilization Committee of Academia Sinica. We aimed to minimize the use of animals used and their suffering without compromising the quality of the experiments.

### EAE induction

Mice were subcutaneously injected in the hind limb with emulsified equal volume of MOG_35-55_ peptide (100 μg/mouse. MDBio, Inc., Taipei) in PBS and complete Freund’s adjuvant (CFA, 400 μg of heat-killed *Mycobacterium tuberculosis* H37RA/mouse, Sigma-Aldrich Co., St. Louis, MO), then intraperitoneally injected with pertussis toxin (PTX, 200 ng/mouse, List Biological Laboratories, Campbell, CA) on the day of immunization and 2 days later. CFA control mice received emulsion without MOG_35-55_ and PTX. As for EAEnp mice, they received the same MOG-CFA emulsion without PTX.

### EAE scoring criteria, analysis parameters and disease phases

The clinical scoring index following a previous report^68^ was assigned daily for 30 days and at the day of behavioral and pathological examination: 0: no clinical signs, 1: paralyzed tail, 2: ataxia of lower body, 3: paraparesis of one or both hind limbs, 4: paraplegia of one or both hind limbs, 5: paraplegia of one or both hind limbs with incomplete paralyzed forelimb, 6: death. Two parameters were used for correlation analysis: peak score, the maximum score within 30 days after induction; accumulated score, summation of all the score in 30 days. For further behavioral experiments, we defined three phases of the EAE disease: pre-onset (day 6 to 7), early onset (day 11 to 12) and recovery (after day 30).

### Mechanical response test

A 0.02-g von Frey filament was applied to each hind paw for 5 times and total withdrawal response was recorded. A positive mechanical response was defined as paw withdrawal, licking or flipping in response to von Frey stimulation. Mechanical response assessment was conducted within 3 days before induction and on selected time points with exclusion of EAE mice with score >3.

### Rota-rod test

The rota-rod test was conducted before EAE induction and on 48 days post-immunization. A rota-rod machine with automatic timers and falling sensors (MK-660D, Muromachi KiKai, Tokyo) were used. In the training session, a constant speed of 4 rpm with 60-sec cut-off time for 3 trials was used. In the test session, the rod accelerated from 4 to 40 rpm with 300-sec cut-off time was used for 3 trials. The longest falling latency was selected to represent the motor function of each mouse.

### Open field test

A transparent square chamber made of plexiglass with area 48 × 48 cm and height 30 cm was used. After 1 hr of habituation in the home cage, mice were allowed to explore the whole test chamber for 20 min and were videotaped for analysis. Top scan software was utilized to analyze the video (Clever System, Reston, VA). The total distance each mouse traveled was analyzed.

### RT-PCR

Mouse spinal cord, sciatic nerve and DRG were freshly collected and stored at −80 °C for further RNA extraction. RNA extraction was performed by using TRIzol reagent (Thermo Fisher Scientific, Waltham, MA). cDNA was synthesized by using non-specific primer Oligo(dT). MOG transcripts were examined by using the following primers: MOG-outer-F: 5′-TGGAAGATCCCTTCTATTGGG-3′, MOG-outer-R: 5′-CTG CCAGTCTTCGGTGCAGCCA-3′, MOG-inner-F: 5′-ATCCTCCTGCAGGTCTC TGT-3′, MOG-inner-R: 5′-CAACCAGGGGTCCAAGAACA-3′, GAPDH-F: 5′-GG AGCCAAAAGGGTCATCATCTC-3′, GAPDH-R: 5′-GAGGGGCCATCCACAGT CTTCT-3′. The MOG outer primers will amplify the MOG coding sequence from 431–709 bp (279 bp) and MOG inner primers will amplify 493–667 bp (175 bp).

### Morphology of axons by transmission electron microscopy

Freshly prepared sciatic nerve was isolated from mice anesthetized with 13% urethane and prefixed in 4% paraformaldehyde with 2.5% glutaraldehyde in 0.1 M sodium Cacodylate buffer (pH 7.2~7.4) overnight. Tissues were immersed in 1% OsO_4_ in s-collidine buffer for post-fixation. Dehydration was performed with ethanol and acetone. Resin infiltration followed increased concentration by 100% acetone: Spurr’s resin (3:1, 1:1, 1:3), each for 2~4 hr and the third step overnight. Tissues were immersed in pure Spurr’s resin for 2 hr or overnight before pure resin for 2~4 hr. After embedding in a mode and polymerization under 60 °C, ultrathin sections 70~90-nm thick were cut by using an ultramicrotome (Leica EM UC7), then mounted on a copper grid. Images were obtained by using FEI Tecnai G2 F20 S-TWIN (FEI, Hillsboro, Oregon). The amount of axon with myelin decompaction or axon-myelin dissociation was counted in 6 randomly selected images in each group.

### Immunohistochemistry

Mice were anesthetized with an intraperitoneal injection of 1.3 mg/kg urethane (Sigma-Aldrich, St Louis, MO, USA) followed by 4% paraformaldehyde fixation (PFA, Merck, Germany). We used 12-μm–thick DRG cryosections and 5-μm–thick spinal cord paraffin-embedded sections for staining. The following primary antibodies were incubated at 4 °C overnight: rabbit anti-ATF3 (1:1000, Santa Cruz Biotechnology, Santa Cruz, CA, USA), rat anti-mouse CD4 (1:100, BD Biosciences, CA, USA), mouse anti-NF200 (1:500, Sigma-Aldrich, St Louis, MO, USA), goat anti-CGRP (1:1000, BioRad, Hercules, CA, USA), rabbit-anti mouse Iba-1 (1:500, Wako Pure Chemical Industry, Osaka, Japan), Alexa Flour 488-conjugated rat anti-mouse GFAP (1:100). Tissue was incubated with secondary antibodies for 1 hr at room temperature: Alexa Fluor 488 donkey anti-goat, goat anti-mouse and goat anti-rabbit; Alexa Fluor 594 donkey anti-rabbit (1:200, Invitrogen technologies, Carlsbad, CA, USA); Alexa Fluor 568 goat anti-rabbit (1:1000); and Alexa Fluor 555 goat anti-rat (1:500, both Thermo Fisher Scientific, Waltham, MA). As for IB4 staining, slides were then immersed in IB4-FITC (Sigma-Aldrich, Inc., St. Louis, MO, USA) containing 1 μM of Mg^2+^, Mn^2+^ and Ca^2+^ for 1.5 hr. Images of DRG sections were acquired under a Ziess Axiovert 200 M fluorescence microscope and processed with ImageJ 64. The maximal background signal was defined as 5 times of the SEM above mean intensity, which is calculated from 9 randomly selected 20 μm × 20 μm square areas from no primary antibody stained images. Images of spinal cord sections were observed by confocal microscopy (LSM 700 stage, Carl Zeiss, Oberkochenm, Germany) and acquired with Zen software (Carl Zeiss, Oberkochen, Germany). Immunofluorescence quantification was analyzed with Integrated Intensity quantification in Metamorph software. To include all possible signals, threshold was set at 70 for Iba-1 staining and 60 for GFAP staining. To further exclude non-specific particles, area limit filter was set at 20 pixel for Iba-1 staining and 50 pixel for GFAP staining. Sections of 3 mice in total were counted in each group.

### H&E and Luxol fast blue (LFB) staining

Spinal cord sections were stained for H&E and LFB as previously described^68^. Briefly, for H&E staining, sections were immersed with Meyer’s hematoxylin followed by 0.5% Eosin Y disodium in 70% alcohol. For LFB staining, sections were stained with 0.1% LFB solution, containing 1 g Solvent blue 38 (Sigma-Aldrich, Inc., St. Louis, MO, USA), 1000 ml 95% alcohol and 5 ml 10% acetic acid, at 60 °C overnight. Photos were obtained by using Pannoramic 250 Flash II (3DHistech Ltd., Budapest, Hungary) and analyzed by panoramic viewer (3DHistech Ltd., Budapest, Hungary).

### Analgesic drug application

The analgesic effects of pregabalin were investigated in the chronic phase of EAE. Following pre-drug assessment, the von Frey test was conducted at 30, 60, 90, 120 and 150 min after drug application. For drug application, pregabalin (Toronto Research Chemicals, Toronto, Canada) was dissolved in normal saline to a stock concentration of 78.5 mM and further diluted with normal saline to a final concentration. Each mouse received pregabalin at doses of 30 or 100 μg/kg or 1 mg/kg intraperitoneally.

### Statistical analysis

Data are presented as mean ± SEM and analyzed by use of Prism 6. Statistical analysis of clinical severity, rota-rod, open-field, mechanical response and drug response test were conducted by repeat measured two-way ANOVA with Bonferroni difference post-hoc testing or Tukey test. One-way ANOVA was used to analyze the activation level of glial cells in the spinal cord and immunofluorescence results. Unpaired *t* test was used to analyze the quantification of axon with axon-myelin association and myelin decompaction. P < 0.05 was considered statistically significant.

## Additional Information

**How to cite this article**: Wang, I-C. *et al*. Peripheral sensory neuron injury contributes to neuropathic pain in experimental autoimmune encephalomyelitis. *Sci. Rep.* **7**, 42304; doi: 10.1038/srep42304 (2017).

**Publisher's note:** Springer Nature remains neutral with regard to jurisdictional claims in published maps and institutional affiliations.

## Supplementary Material

Supplementary Information

## Figures and Tables

**Table 1:** Spearman correlation analysis of pain levels verses peak scores or cumulative scores at 30–39 and 40–49 days post-induction in wild-type, *Asic1a* ^−/−^, *Asic2* ^−/−^ and *Asic3* ^−/−^ EAE mice.

	D30-39				D40-49			
	Wildtype	*Asic1a*^−/−^	*Asic2*^−/−^	*Asic3*^−/−^	Wildtype	*Asic1a*^−/−^	*Asic2*^−/−^	*Asic3*^−/−^
	**Max. score**							
Spearman r	−0.08778	−0.02700	0.2722	0.1248	0.1077	−0.3021	0.08944	−0.06561
95% CI	−0.3826 to 0.2233	−0.6294 to 0.5957	−0.3743 to 0.7406	−0.2870 to 0.4977	−0.1970 to 0.3934	−0.7720 to 0.3813	−0.5248 to 0.6424	−0.4313 to 0.3186
p value	0.5710	0.6190	0.6636	0.5436	0.4764	0.1498	0.3727	0.7353
Significance	ns	ns	ns	ns	ns	ns	ns	ns
	**Cumulative score**							
Spearman r	0.1566	0.1541	0.1539	0.003135	0.1892	−0.1125	−0.1986	−0.2094
95% CI	−0.1559 to 0.4407	−0.5066 to 0.7008	−0.4758 to 0.6793	−0.3949 to 0.4002	−0.1157 to 0.4615	−0.6785 to 0.5374	−0.7033 to 0.4393	−0.5430 to 0.1811
p value	0.3099	0.6529	0.6494	0.9879	0.2080	0.6680	0.4043	0.2755
Significance	ns	ns	ns	ns	ns	ns	ns	ns
