# Supplementary material for: Peripheral sensory neuron injury contributes to neuropathic pain in experimental autoimmune encephalomyelitis
Source: Sci Rep. 2017 Feb 9;7:42304. doi: 10.1038/srep42304 (PMC5299449; doi:10.1038/srep42304)
Supplement: Supplementary Information [file srep42304-s1.pdf]

Peripheral sensory neuron injury contributes to neuropathic pain in experimental autoimmune encephalomyelitis

I-Ching Wang, Chen-Yen Chung, Fang Liao, Chih-Cheng Chen, Cheng-Han Lee

Supplementary methods

Western blot

After mice sacrificed, total DRG were freshly collected in ice-cold homogenized buffer, which contains 50mM Tris-HCl (pH7.4), 120mM sodium chloride, 1mM EDTA, 5mM potassium chloride and 4mM magnesium chloride. After homogenized, the homogenates were centrifuged at 200 xg, 10min, 4°C to remove cell debris. The supernatant was collected and centrifuged at 48000 xg, 20min, 4°C. After centrifugation, the supernatant containing the cytosolic proteins was saved and the pellet representing the membrane fraction was resuspended in 300 μl lysis buffer containing 1% digitonin, 50mM Tris-HCl, 300mM NaCl, and 100mM PMSF for 1 hour at 4°C. 10μg of each sample was loaded in 10% acrylamide gel for electrophoresis followed by transferring to PVDF membrane (PerkinElmer, Taipei, Taiwan). Membrane was incubated in 5% skimmed milk in PBS with 0.1% tween-20 (PBS-T) for 1 hour, room temperature. All primary antibodies were incubated at 4°C overnight. ASIC1a was stained by Goat anti-ASIC1 (1:200, Santa Cruz Biotechnologies Inc., Texas, USA) antibody, the membrane and cytosol markers used were Mouse anti- α1-sodium potassium ATPase (1:1000, Abcam, Cambridge, UK)

and Mouse anti-α-tubulin (1:5000, Sigma-Aldrich, St Louis, MO, USA) respectively. Anti-goat and anti-mouse secondary antibodies conjugated with HRP were incubated for 1 hour at room temperature. Chemifluorescence signal was captured and quantified by UVP biospectrum auto imaging system (UVP, Upland, CA). ASIC1a level of membrane fraction was normalized by α-tubulin.

Hargreaves test

Thermal pain was conducted with Hargreaves machine (IITC Inc. Life Science, CA). The radiant heat stimulus intensity was set as 20% and the inactive intensity was set as 5%. Mice were allowed to habituate for at least 30 minutes before experiment. Every mouse was tested 3 times per each hind paw. Thermal response assessment was performed within 3 days prior to EAE induction and selected time points. EAE mice with score higher than 3 were excluded.

Quantitative PCR

Total RNA was isolated from lumbar part dorsal root ganglia with TRIzol reagents and transcribed into cDNA with non-specific primer Oligo(dT). Next, quantitative

PCR was performed on ABI prism 7500 Sequence Detection system (Thermo Fisher Scientific, Waltham, MA). Gene expression levels were evaluated and quantified by using Taqman probes of GAPDH (Mm99999915_g1), IL17a (Mm00439618_m1) and IFNγ (Mm01168134_m1). Each amplification reaction was performed in duplicate and carried out in a 20-μl reaction, containing 100ng cDNA, 1μl of 20X Taqman probe, and 10μl of 2X Taqman Universal PCR Master Mix. The reactions were completed as follows: 2 min at 50°C (1 cycle); 10 min at 95°C (1 cycle); 15 s at 95°C, and 60 s at 60°C (65 cycles). Gene expression was detected using the $\Delta\Delta C_T$ method relative to GAPDH and normalized to naïve treatment.

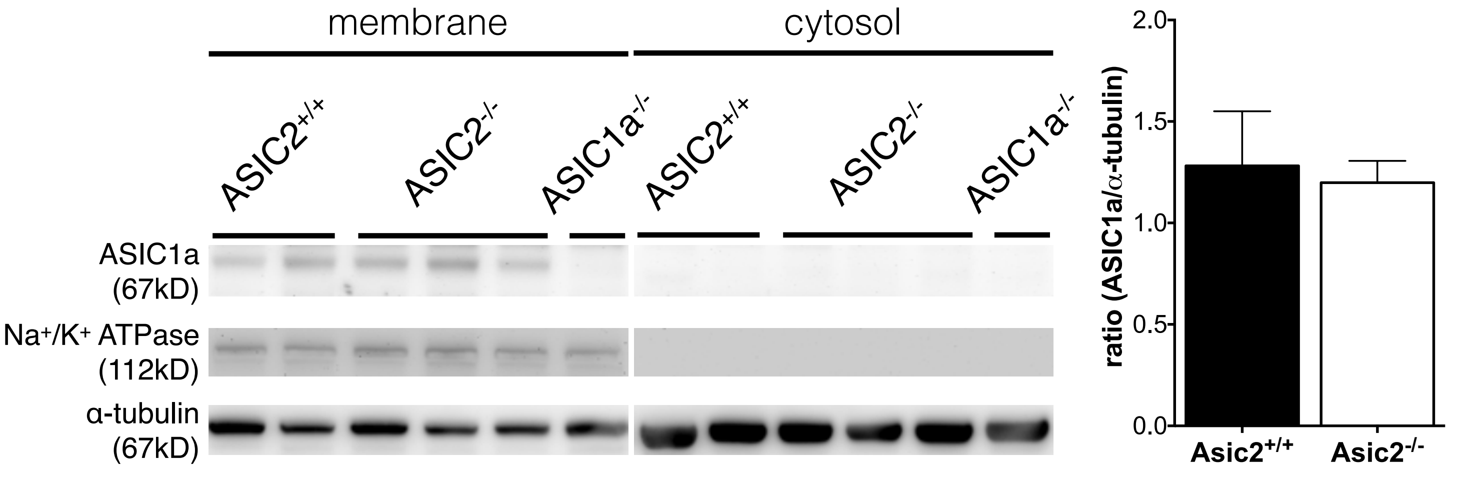

Supplementary Figure S1. Expression of ASIC1a in DRG of ASIC2 knockout mice. Total DRGs were collected to detect ASIC1a protein level. Na+/K+ ATPase is a membrane protein for sample normalization to total membrane fraction and α-tubulin was a cytosolic marker. DRG of *Asic1a*$^{-/-}$ was a negative control for ASIC1a signal. *Asic2*$^{+/+}$ N=2, *Asic2*$^{-/-}$ N=3 for quantitative analysis. t test P=0.7544.

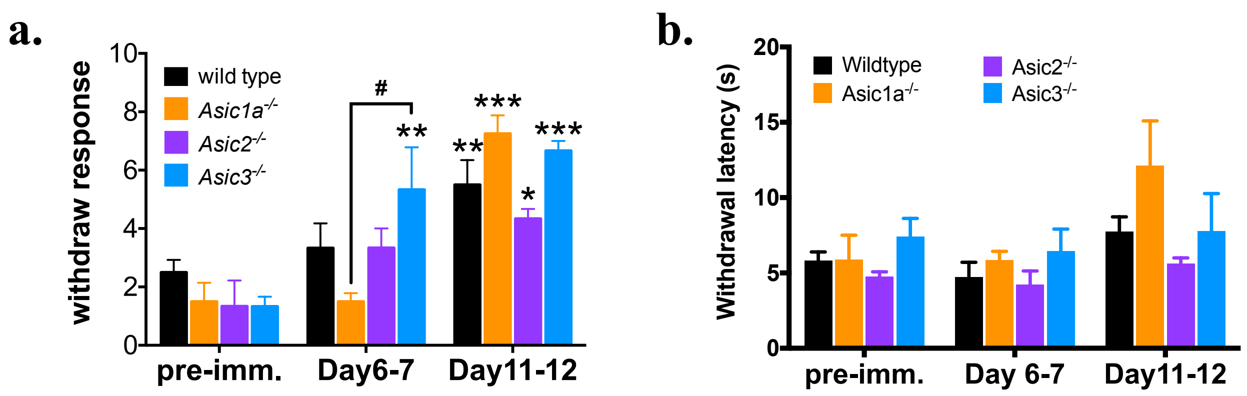

Supplementary Figure S2. Mechanical and thermal responses of ASIC-subtype knockout mice in pre-onset and early onset phases. **a.** Mechanical responses to von Frey test. Two-way ANOVA, Time : $F_{(2, 24)} = 34.00$, $P<0.0001$, treatment : $F_{(3, 12)} = 1.266$, $P=0.3301$ interaction : $F_{(6, 24)} = 3.062$, $P=0.0227$, * $P<0.05$, ** $P<0.01$, *** $P<0.001$ compared with respective pre-immune group. # $P<0.05$. **b.** Thermal sensitivity assessment by Hargreaves test. Two-way ANOVA, interaction: $F_{(6, 24)} = 1.060$, $P = 0.4133$; Time: $F_{(2, 24)} = 4.969$, $P = 0.0156$; Genotype: $F_{(3, 12)} = 2.320$, $P = 0.1270$. Wild-types N=6; *Asic1a*$^{-/-}$ N=4, *Asic2*$^{-/-}$ N=3, *Asic3*$^{-/-}$ N=3.

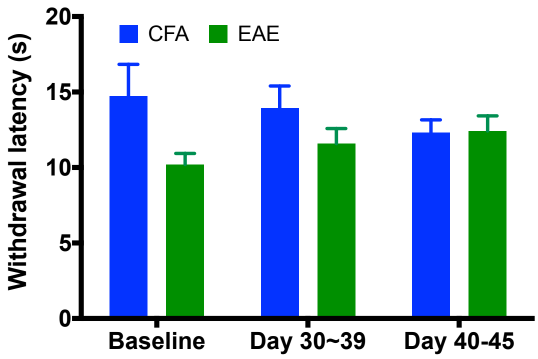

Supplementary Figure S3. EAE mice showed no difference in thermal hyperalgesia compared with CFA mice in the recovery phase. Two-way ANOVA, interaction: $F_{(2,\ 18)} = 1.675$, $P = 0.2152$; Time: $F_{(2,\ 18)} = 0.052$, $P = 0.9495$; Treatment: $F_{(1,\ 9)} = 5.023$, $P = 0.0517$. CFA:N=3, EAE: N=8.

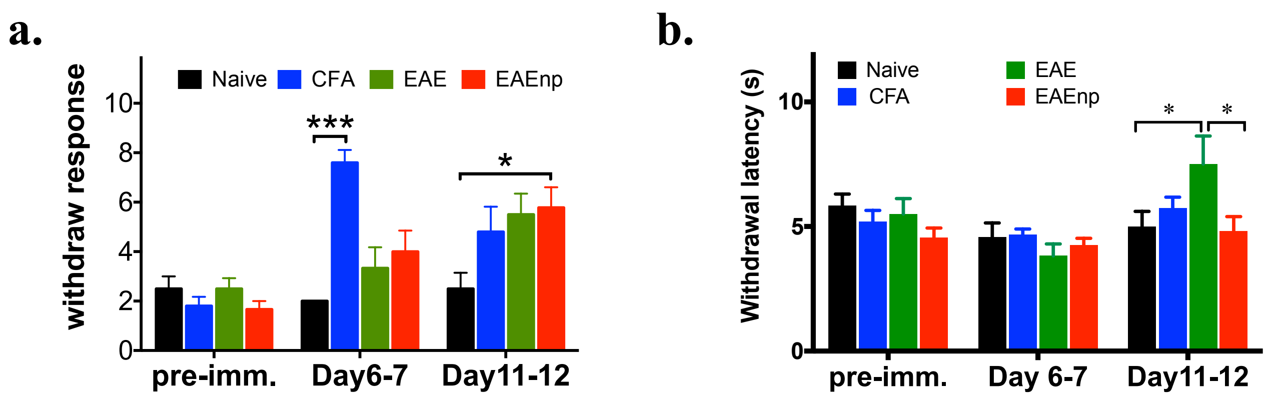

Supplementary Figure S4. Mechanical and thermal response of Naive, CFA, EAE and EAEnp mice in pre-onset and early onset phases. **a.** Mechanical response by von Frey test. Two-way ANOVA, time: $F_{(2, 40)}$=14.63, P<0.0001; treatment: $F_{(3, 20)}$=3.068, P=0.0514; interaction: $F_{(6, 40)}$=4.764, P=0.001 *: P<0.05, ***: P<0.001 compared with naive control in the same time point. **b.** Thermal sensitivity examination by Hargreaves test. Two-way ANOVA, interaction: $F_{(6, 40)}$ = 2.136, P = 0.0702; time: $F_{(6,40)}$ = 6.435, P = 0.0038; treatment: $F_{(3, 20)}$ = 2.130, P = 0.1284. Naive: N=4, CFA: N=5, EAE: N=6, EAEnp: N=9.

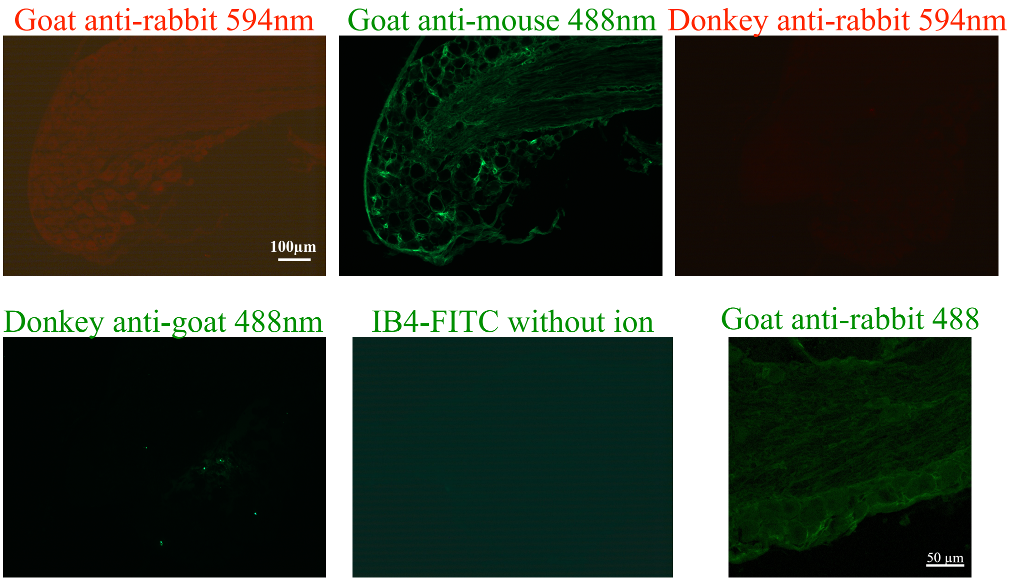

Supplementary Figure S5. Secondary antibody controls for immunohistochemistry staining on wild-type DRG sections.

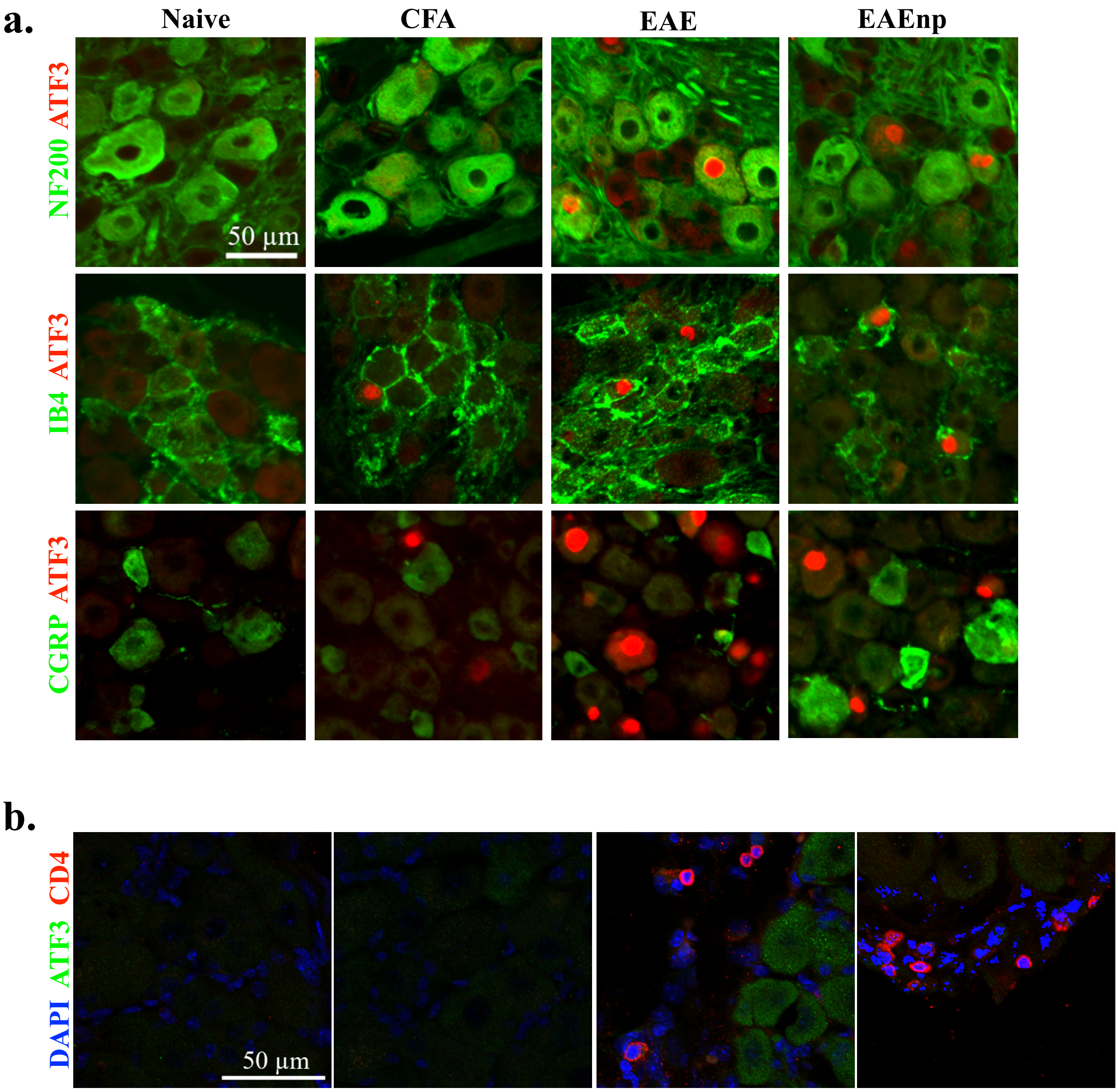

Supplementary Figure S6. Magnified images of Fig 6.

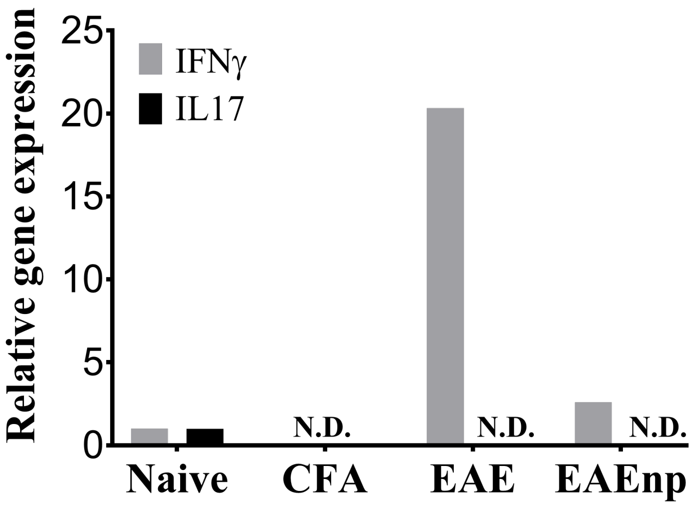

Supplementary Figure S7. Quantitative PCR results of interferon γ and interleukin 17 in lumbar DRG of naive, CFA, EAE and EAEnp mice. mRNA level of IFNγ and IL-17 is relative to GAPDH and normalized to naive. N=1 in each group, N.D. not detected
